# A new route to enhance the ferromagnetic transition temperature in diluted magnetic semiconductors

**DOI:** 10.1038/s41598-017-09729-6

**Published:** 2017-08-29

**Authors:** Kalpataru Pradhan, Subrat K. Das

**Affiliations:** 1 CMP Division, Saha Institute of Nuclear Physics, HBNI, Kolkata, 700064 India; 2 SKCG Autonomous College, Paralakhemundi, Odisha 761200 India

## Abstract

We investigate the magnetic and the transport properties of diluted magnetic semiconductors using a spin-fermion Monte-Carlo method on a simple cubic lattice in the intermediate coupling regime. The ferromagnetic transition temperature *T*_c_ shows an optimization behavior with respect to the absolute carrier density *p*_abs_ and the magnetic impurity concentration *x* as seen in the experiments. Our calculations also show an insulator-metal-insulator transition across the optimum *p*_abs_ where the *T*_c_ is maximum. Remarkably, the optimum *p*_abs_ values lie in a narrow range around 0.11 (holes/site) for all *x* values and the ferromagnetic *T*_c_ increases with *x*. We explain our results using the polaron percolation mechanism and outline a new route to enhance the ferromagnetic transition temperature in experiments.

## Introduction

Diluted magnetic semiconductors (DMS) are materials of strong interest due to both, their novel ferromagnetism and potentiality for future spintronics^1–5^. In particular, Ga_1−*x*_Mn_*x*_As^6, 7, 8﻿^ with ferromagnetic transition temperature *T*_c_ $\simeq$ $\simeq$191 K in films^9^ and even more ($\simeq$200 K)^10^ in nano wires has led intensive efforts to increase *T*_c_ in view of possible technological applications.

It is widely accepted that S = 5/2 Mn^2+^ ion replaces Ga^3+^ ion in Ga_1−*x*_Mn_*x*_As and thereby contributes a hole to the semiconductor valence band, which mediate the magnetic interaction between the localized spins. However, point defects like Mn interstitials (Mn_I_) and As antisites^11, 12^ significantly compensate the free hole density. In addition, Mn_I_ are highly mobile and preferentially choose the interstitial positions adjacent to Ga substituted Mn ions (Mn_Ga_), thus forming anti-ferromagnetic Mn_Ga_ − Mn_I_ pairs^13^ and consequently increases the Mn inactive sites. So, overall Mn_I_ reduces the hole density and the effective Mn concentration (*x*_eff_) to Mn_Ga_-2Mn_I_ and Mn_Ga_-Mn_I_, respectively, hindering the higher ferromagnetic *T*_c_ in (Ga,Mn)As. We define the carrier density *p* = *p*_abs_/*x*, where *x* is the Mn concentration and *p*_abs_ is the absolute carrier density. So in this paper *p*_abs_ denotes *holes per each site*, while *p* denotes *holes per each impurity site*. The *p*_abs_ in our language is similar to the hole density (holes per cm^3^) generally reported in the experiments.

Most of the experimental studies have been devoted in the search of room-temperature ferromagnetism using different methods. Post-growth annealing is one of the most extensively used technique which enhances the *T*_c_ by reducing the Mn_I_ concentration and in turn increasing the carrier concentration^14^. It is important to note that all Mn_I_ can not be removed from the sample^15^. Even after the annealing 20% of the Mn impurities remain at the interstitial positions for *x* ≈ 0.1, putting a limit to the *T*_c_ which either saturates or decreases at larger *x* ^16, 17^. Consequently, the system goes from insulating to metallic and then again to insulating phase with *x* ^18^.

Another route to enhance the *T*_c_ is by co-doping *p*-type or *n*-type dopants that can tune the hole density. The enhancement of *T*_c_ depends upon *x* and type of the co-dopant. It is observed that Be (*p*-type co-dopant) co-doping in Ga_1−*x*_Mn_*x*_As increases both the hole density and the *T*_c_ for magnetic impurity concentration *x* = 0.03 [ref. 19]. On the other hand, for *x* = 0.05 the hole density either saturates or decreases due to the increase in Mn_I_ concentration and as a result *T*_c_ decreases^19, 20^. In contrast, Si (*n*-type co-dopant) co-doping decreases the hole density for all values of *x* [refs 21 and 22]. In this case, for *x* ≤ 0.08 *T*_c_ decreases as compared to the un-codoped ones, but for higher *x* (>0.08) Si co-doping increases the *T*_c_. Now, if we focus on the enhancement of *T*_c_ and summarize the experimental results then in the low impurity concentration regime (*x* ≤ 0.08) if the hole density increases, with co-doping, the ferromagnetic *T*_c_ increases. And, in the higher impurity concentration regime the ferromagnetic *T*_c_ increases with decrease in the hole density as compared to the un-codoped samples.

It is believed that it is necessary to increase effective Mn concentration to enhance the *T*_c_^8^, but *T*_c_ decreases beyond *x*_eff_ = 0.07 [ref. 17]. So it is important to search a route in which both the Mn concentration and the hole density can be altered using growth and post-growth techniques. In this report, we investigate this scenario and outline a procedure to enhance *T*_c_ with Mn impurity concentration *x*. We calculate the ferromagnetic *T*_c_ within a diluted Kondo lattice model in the intermediate coupling regime using a Monte-Carlo technique based on travelling cluster approximation^23^ on large size systems. The ferromagnetic *T*_c_ shows an optimization behavior with *x* and *p*_abs_, and in the process system undergoes an insulator-metal-insulator transition. Our results qualitatively agree with the recent experiments. We find that optimum *p*_abs_, where *T*_c_ is maximum, lies around 0.11 for a wide range of *x* = 0.1–0.35. And, for a fixed *p*_abs_ ferromagnetic *T*_c_ increases with *x*, which suggests a new pathway to achieve high temperature DMS.

## Model Hamiltonian

We consider a diluted Kondo lattice Hamiltonian^24–26^ on a simple cubic lattice:

$$H=-t\,\sum _{\langle ij\rangle \sigma }\,({c}_{i\sigma }^{\dagger }{c}_{j\sigma }+h.c\mathrm{.)}-\frac{J}{2}\,\sum _{R}\,{{\bf{S}}}_{R}.{\vec{\sigma }}_{R}-\mu \,\sum _{i}\,{c}_{i\sigma }^{\dagger }{c}_{i\sigma },$$

where ${c}_{i\sigma }^{\dagger }$ (*c*_*iσ*_) are the fermion creation (annihilation) operators at site *i* with spin *σ* and *t* is the nearest neighbor (〈*ij*〉) hopping parameter. The second term represents the Hund’s coupling *J* between the localized impurity spin **S**_*R*_ and the itinerant electrons ${\vec{\sigma }}_{R}$ (represented by Pauli spin matrices) at random site *R*. The itinerant electrons mediates an indirect interaction between the localized spins **S**_*R*_. As the magnetic moment *S*_*R*_ = 5/2, in this paper, we considered the spin *S*_*R*_ to be classical and absorb the magnitude of *S*_*R*_ into *J* without loss of generality. *μ* is the chemical potential. Magnetic moment clustering and hence the direct exchange interaction between the localized spins are neglected. In our particle-hole symmetric model the magnetic and transport properties are presented in terms of fixed hole density. In order to get the same hole density throughout the calculations (hole density checked using Fermi-Dirac distribution) we tune the chemical potential *μ* during the annealing process at each temperature. *J* is scaled with hopping parameter *t*. Using a realistic bandwidth W = 12*t* = 6 eV for the host semiconductor GaAs we set *t* = 0.5 eV.

We employ exact diagonalization based Monte-Carlo (ED + MC) approach to anneal the system towards the ground state at fixed density and temperature. In this method the classical spin **S**_*R*_ is updated at a site and the internal energy is calculated by exact diagonalization of the carriers in the background of the new spin configuration. The proposed update is accepted or rejected by using the classical Monte-Carlo scheme based on Metropolis algorithm. A single system sweep constitutes the above process repeated over each classical spin once. At each temperature one needs at least over 2000 system sweeps to anneal the system sufficiently. But, the exact diagonalization is numerically expensive and grows as O(N^4^) per system sweep where *N* is the number of lattice sites. So, we employ travelling cluster approximation (TCA)^23, 27^ to handle system size as large as *N* = *L* ^3^ = 10^3^. In TCA, the Monte-Carlo scheme is implemented by diagonalizing a Hamiltonian reconstructed from a cluster around the to-be-updated site rather than diagonalizing the full lattice. The cost of computation for system sweep reduces to O($N\times {N}_{{\rm{c}}}^{3}$) for a cluster size *N*_c_. We use a cluster size *N*_c_ = 4^3^ in our calculations. All physical quantities are averaged over ten different randomly localized spin configurations.

## Results and Discussion

**Figure 1:**
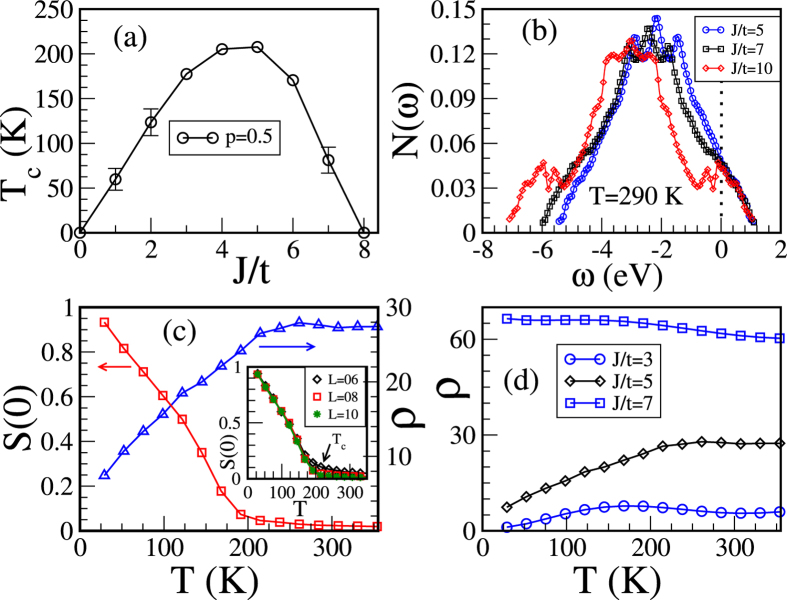
(**a**) Shows optimization behaviour of ferromagnetic *T*_c_ with *J*/*t* for fixed *p* = 0.5; (**b**) density of states *N*(*ω*) showing the formation of an impurity band for *J*/*t* = 7 and 10 at *T* = 290 K. Fermi energy is set at zero; (**c**) temperature dependence of the ferromagnetic structure factor *S*(**0**) and resistivity in units of *ħa*/*πe* ^2^ (*a* is lattice constant) for fixed *J*/*t* = 5 and *p* = 0.5. This shows the onset of ferromagnetism and metalicity at the same temperature. Inset: ferromagnetic structu﻿re factor *S*(**0**) vs temperature for three different lattice sizes *L* = 6, 8, and 10 where *T*_c_ (indicated by arrow) is hardly distinguishable for *L* = 8 and 10; (**d**) temperature dependence of the resistivity (in units of *ħa*/*πe* ^2^) for *J*/*t* = 3, 5, and 7 showing a metal-insulator transition with increasing *J*/*t*.

We start our calculations for different *J* values with a specific choice of *x* = 0.25 and *p* = 0.5 which is a good starting point for simple cubic lattice. A simple cubic lattice has one atom per unit cell, where as GaAs is face centered cubic with four atoms per unit cell. So, roughly 25% of *x* in our case corresponds to 6.25% that in real experiments. Magnetic and transport properties in DMS are the consequence of the competition between the carrier mediated ferromagnetic spin-spin interaction and the carrier localization. For *J* ~ 0 there is no carrier mediated spin-spin interaction and as a result there is no ferromagnetism. A minimum value of *J* is required to generate the ferromagnetic interaction which also depends upon *x* and *p*. As *J* increases from a smaller value ferromagnetic ordering starts to develop. Further, at larger *J* the carriers get trapped in spin impurity sites and the ferromagnetism is suppressed. So the optimal *T*_c_ lies in the intermediate range of *J* as shown in Fig. 1(a). The carrier localization for higher *J* is apparently clear from the the developments of an impurity like band in the density of states $N(\omega )=\langle \tfrac{1}{N}{\sum }_{\alpha }\,\delta (\omega -{\varepsilon }_{\alpha })\rangle$ at relatively high temperature *T* = 290 K as shown in Fig. 1(b). The ferromagnetic *T*_c_ is estimated from the spin-spin correlation function *S*(**q**) = $\tfrac{1}{N}{\sum }_{ij}\,{{\bf{S}}}_{{\bf{i}}}\cdot {{\bf{S}}}_{{\bf{j}}}{e}^{i{\bf{q}}\cdot ({{\bf{r}}}_{{\bf{i}}}-{{\bf{r}}}_{{\bf{j}}})}$, where the ferromagnetic order is indicated by a peak at wave vector **q** = **0**. In Fig. 1(c) we plot the ferromagnetic structure factor *S*(**0**) vs temperature for *J*/*t* = 5, *x* = 0.25, and *p* = 0.5. The inset shows that *S*(**0**) for *L* = 8 and *L* = 10 are barely distinguishable at *T*_c_. So we have considered *L* = 8 for rest of our calculations.

We obtain the resistivity for different *J* values by calculating the *dc* limit of the conductivity determined by the Kubo-Greenwood formula^28, 29^ as shown in Fig. 1(d). At low temperature the system shows metallic behavior for small and intermediate *J* values. As *J* increases (*J*/*t* = 7) the system remains insulating in the whole temperature range due to carrier localization at impurity sites. For rest of our calculations we use intermediate coupling strength *J*/*t* = 5 where *T*_c_ is found to be maximum and relevant to DMS. Temperature dependence of the ferromagnetic structure factor and the resistivity [see Fig. 1(c)] show one-to-one correspondence between the onset of the ferromagnetism and the metalicity at *T*_c_ ~ 200 K.

**Figure 2:**
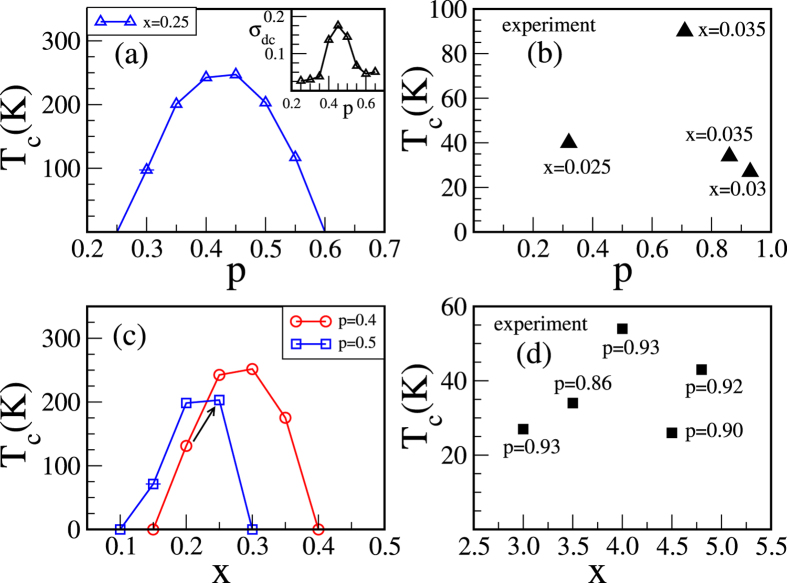
For fixed *J*/*t* = 5: (**a**) pl﻿ots *p* dependence of the ferromagnetic *T*_c_ and (inset) the *dc* conductivity in units of *πe* ^2^/*ħa* calculated at T = 29 K for fixed *x* = 0.25. It shows the correlation between the ferromagnetic *T*_c_ and the carrier mobility; (**c**) variation of the ferromagnetic *T*_c_ with *x* for *p* = 0.4 and 0.5. The arrow mimics the effect post-growth annealing on *T*_c_; (**b**,**d**) shows the experimental results (ref. 17) on ferromagnetic *T*_c_ vs *p* and *x*, respectively.

In carrier-mediated magnetic systems a minimum amount of carrier is essential to initiate the coupling between the magnetic spins, which depends on *J*/*t* and *x*. On the other hand, for higher carrier density the magnetism is suppressed due to decrease in carrier mobility. The overall behavior of *T*_c_ with *p* is shown in Fig. 2(a) for *J* = 5 and *x* = 0.25. The mobility picture is confirmed from the conductivity calculation, where *T*_c_ and the conductivity (at the low temperature) are maximized at *p* = 0.45 (see the inset). In order to compare our result with the experiment we plot the data from ref. 17 in Fig. 2(b) such that the impurity concentrations *x* lie in a narrow range from 0.025–0.035 which we assume to be nearly constant. Now, if we match, the *T*_c_ vs *p* behavior in the experiment is very similar to our results. A metal-insulator transition with *p* is also observed in the same experiment (not shown here) which we already illustrated the inset of Fig. 2(a).

The carrier mobility and hence the ferromagnetism can be tuned with *J*, *p* or *x* independently. The carrier-spin interaction *J* is only operative at the impurity sites i.e. for fixed *J* value the effective coupling strength of the system increases with impurity concentration *x*. This is similar to the case of increasing *J* with keeping *x* fixed. So the variation of *T*_c_ with *x* for fixed *p* values [Fig. 2(c)] can be understood from the *T*_c_ dependence of *J*/*t* as in Fig. 1(a). We plot the experimental data from the ref. 17 in Fig. 2(d) such that the carrier densities *p* lie in a narrow range from 0.86–0.93. We have neglected this small variation of *p* for qualitative comparison with our calculations and find that the *T*_c_ shows an optimization behavior with *x*, quite similar to our results. It is important to note that if we increase both *x* and *p* along the arrow shown in Fig. 2(c) the *T*_c_ increases, which mimics the effect of post-growth annealing on *T*_c_. Our calculated *T*_c_ values in Fig. 2(a,c) are higher than the experimental *T*_c_ values [in Fig. 2(b,d)] due to the higher impurity concentrations used in our calculations.

**Figure 3:**
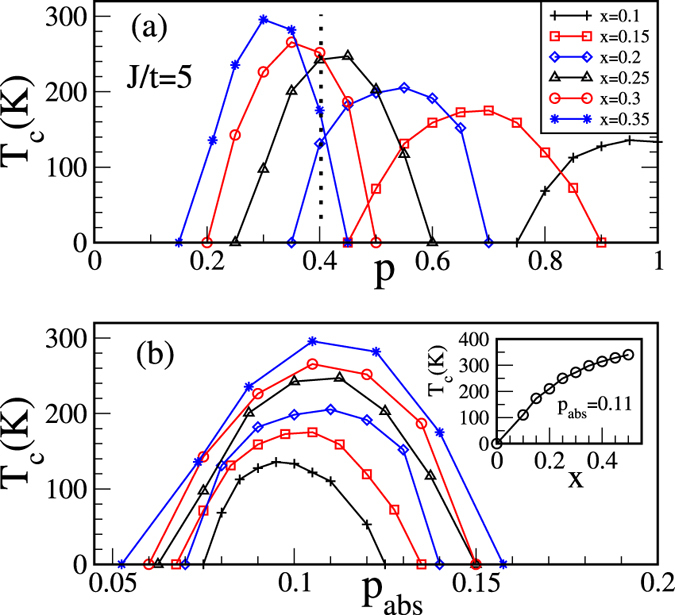
Ferromagnetic windows in terms of the ferromagnetic *T*_c_ vs (**a**) the hole density *p* and (**b**) the absolute hole density *p*_abs_ for different impurity concentrations *x* for fixed *J*/*t* = 5. The symbols for different *x* values in (**b**) are the same as in (**a**). Inset: *x* dependence of ferromagnetic *T*_c_ for fixed *p*_abs_ = 0.11 showing an increase in *T*_c_ with *x*.

Figure 3(a) shows the ferromagnetic windows for various values of *x* in a wide range starting from as small as 10%. We find that the optimal *p* value where the *T*_c_ is maximum decreases with *x*, which is in contrast to the earlier claim where *p* = 0.5 is suggested to be the optimum value irrespective of impurity concentrations^24^. Further, our results explain the experimental findings where both *x* and *p* can be changed simultaneously. In experiments, it is observed if *p* decreases with co-doping then for high (low) impurity concentration *x* the ferromagnetic *T*_c_ increases (decreases)^21, 30^. To compare our results with the experiment we focus around *p* = 0.4, the dotted line in Fig. 3(a). Now, if we decrease *p* the transition temperature decreases for lower values of *x* (=0.25 and 0.20) but increases for higher values like *x* = 0.30 and 0.35, which captures the experimental results discussed above. Our calculations clearly demonstrate that *T*_c_ can be increased with *x* provided the *p* value is tuned properly but not arbitrarily. For *x* = 0.1 and *t* = 0.5 eV the estimated *T*_c_ is 120 K, which matches reasonably well with the experiments^18^.

It is generally believed that the *p* value must be maximized to obtain a higher ferromagnetic *T*_c_ in DMS. In Fig. 3(a) our calculations show otherwise, the optimum *p* value decreases with increasing *x*. To interpret our finding, in Fig. 3(b), we re-plot the ferromagnetic windows in terms of the absolute carrier density *p*_abs_ as defined earlier. Interestingly, we find that the ferromagnetic windows lie on top of each other with optimum *p*_abs_ around 0.11 for *x* = 0.35, which decreases slightly for smaller *x* values. To understand this we start our discussion from the double exchange (large *J*) limit where carrier spins are aligned in the direction of the core spin. For *x* = 1 (spins at each site) carriers get delocalized and the electronic kinetic energy is minimized for the ferromagnetic ground state in the range 0 < *p*_abs_ < 1, where the optimum ferromagnetic *T*_c_ is found to be at *p*_abs_ = 0.5 [ref. 31]. This we call the optimum *p*_abs_. The range of ferromagnetic ground state is confined to 0 < *p*_abs_ < 0.3 in the intermediate coupling regime and the optimum *p*_abs_ decreases to ~0.15 [ref. 32]. In the diluted limit (*x* = 0.1–0.35) we find [see Fig. 3(b)] that the optimum *p*_abs_ value is ~0.11 which is in the right ball park as compared to the *x* = 1 limit. This can be understood within a polaron picture discussed below.

**Figure 4:**
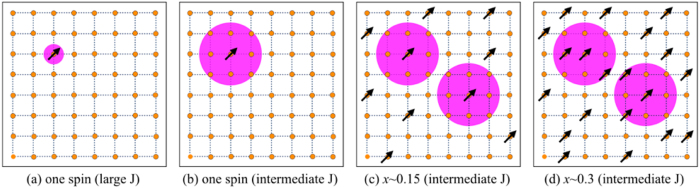
2D Schematics show the electron delocalization picture by the shaded regions (polarons) for four different cases (arrow indicate the impurity spin); (**a**) localized polaron in the double exchange limit (large *J*/*t*) and (**b**) extended polaron in intermediate coupling regime for one spin and one electron case; extended polarons are shown in the intermediate coupling regime for two different diluted limits (**c**) *x* = 0.15 and (**d**) *x* = 0.3.

In the double exchange limit for one spin and one carrier problem the carrier remains localized to the core spin. A single site localized polaron is shown schematically as the shaded region in Fig. 4(a). In the intermediate coupling regime the carrier delocalization extends over many lattice sites as shown in Fig. 4(b). For a given *x* a minimum concentration of polarons is required for ferromagnetic percolation. For *x* = 0.15 the ferromagnetic window starts at *p*_abs_ $\simeq$ 0.07 and *T*_c_ is maximum for *p*_abs_ $\simeq$ 0.10. When we increase *x* the optimum *p*_abs_ does not change in the range *x* = 0.1–0.35, studied in this paper. This indicates that the number of spins in the shaded region increases without affecting the polaron size, schematically shown in Fig. 4(c,d). Now, if we plot *T*_c_ vs *x* for *p*_abs_ = 0.11 then *T*_c_ increases with *x* in the diluted limit and saturates for concentrated *x* values [see the inset of Fig. 3(b)].

Using the insight obtained from our calculations we suggest a two step procedure to enhance the ferromagnetic *T*_c_ in experiments. The first step is to determine the optimum carrier density *p*_abs_ for a fixed impurity concentration *x*. So in this step one needs to tune *p*_abs_ without changing *x*, which can be achieved by using an external process like hydrogenation^33, 34^. After extracting the optimal *p*_abs_ the second step is to increase only *x* without altering the optimal *p*_abs_ value obtained in the first step. With increasing *x* the *p*_abs_ would change too, which can be tuned back to its optimal value by co-doping with suitable (n-type or p-type) element. It is important to note that co-doping not only tunes the hole density but also increases the effective *x* ^35, 36^ and will be helpful to enhance the *T*_c_ further. We believe that a systematic combination of experimental processes *e.g.* doping, annealing, hydrogenation, and co-doping can be designed to prepare DMS with higher *T*_c_.

## Conclusion

In conclusion, our model calculations provide a new framework to increase the ferromagnetic *T*_c_ in diluted magnetic semiconductors. The optimum *p*_abs_ (absolute carrier density), where *T*_c_ is maximum, lies around 0.11 and *T*_c_ increases with *x* for fixed *p*_abs_ in a broad range of *x* studied in this letter. To replicate such a scenario in the experiment, *p*_abs_ has to be determined for small *x* and then effort should be made to increase *x* without altering the *p*_abs_ value. This procedure, viable in experiments, would enhance the ferromagnetic *T*_c_. We hope that our finding will motivate new experiments by combining the growth and the post-growth process outlined here to achieve high *T*_c_ DMS for spintronics applications.
